# Seronegative Sicca Syndrome: Diagnostic Considerations and Management Strategies

**DOI:** 10.3390/life15060966

**Published:** 2025-06-17

**Authors:** Yordanka M. Basheva-Kraeva, Krasimir I. Kraev, Petar A. Uchikov, Maria I. Kraeva, Bozhidar K. Hristov, Nina St. Stoyanova, Vesela T. Mitkova-Hristova, Borislav Ivanov, Stanislav S. Karamitev, Nina Koleva, Aleksandar Marinkov, Veselin A. Vassilev

**Affiliations:** 1 Department of Ophthalmology, Faculty of Medicine, Medical University of Plovdiv, 4002 Plovdiv, Bulgaria; dannybasheva@gmail.com (Y.M.B.-K.); nina.st.st@abv.bg (N.S.S.); vesela_mitkova@abv.bg (V.T.M.-H.); 2 Department of Propedeutics of Internal Diseases, Medical Faculty, Medical University of Plovdiv, 4002 Plovdiv, Bulgaria; marinkov.aleksandar@yahoo.com; 3 Department of Special Surgery, Medical Faculty, Medical University of Plovdiv, 4002 Plovdiv, Bulgaria; puchikov@yahoo.com; 4 Department of Otorhinolaryngology, Medical Faculty, Medical University of Plovdiv, 4002 Plovdiv, Bulgaria; kraevamaria93@gmail.com; 5 Second Department of Internal Diseases, Section “Gastroenterology”, Medical Faculty, Medical University of Plovdiv, 4002 Plovdiv, Bulgaria; hristov.bozhidar@abv.bg; 6 Faculty of Medicine, Medical University of Plovdiv, 4002 Plovdiv, Bulgaria; borislav.j.ivanov@gmail.com; 7 Department of Orthopedics and Traumatology, Faculty of Medicine, Medical University of Plovdiv, 4002 Plovdiv, Bulgaria; dokstanly@yahoo.com; 8 Speciality “Assistant Pharmacist”, Medical College, Medical University of Plovdiv, 4002 Plovdiv, Bulgaria; nina.koleva@mu-plovdiv.bg; 9 Department of Physiology, Medical Faculty, Medical University of Plovdiv, 4002 Plovdiv, Bulgaria; veselin.vasilev@mu-plovdiv.bg

**Keywords:** seronegative sicca syndrome, primary Sjögren’s syndrome, autoimmune exocrinopathy

## Abstract

Seronegative sicca syndrome encompasses patients who present with xerostomia and/or keratoconjunctivitis sicca but lack anti-SSA/SSB antibodies and do not fulfill current classification criteria for primary Sjögren’s syndrome (pSS). Despite symptom overlap with pSS, these individuals remain diagnostically and therapeutically unclassified. This review studies the clinical, immunological, and pathological spectrum of seronegative sicca, highlighting its heterogeneity and the limitations of antibody-centric diagnostic frameworks. Histopathologic findings in some seronegative patients—including focal lymphocytic sialadenitis—mirror those seen in pSS, suggesting underlying immune-mediated glandular damage. In others, nonspecific or normal biopsy findings suggest non-immune mechanisms. New evidence of immune activity, such as elevated cytokines (BAFF, IFN-α), and novel autoantibodies (SP-1, CA-VI), further supports the concept of subclinical autoimmunity in a subset of these patients. Clinically, they often face significant burden, including dryness, fatigue, and pain, yet remain excluded from most research cohorts, therapeutic trials, and clinical guidelines. Their management is often individualized, relying on symptomatic therapies rather than immunomodulatory agents. The lack of validated diagnostic criteria and prognostic markers compounds the uncertainty surrounding disease evolution, as some patients may later seroconvert or develop systemic features. To address these gaps, a paradigm shift is needed—one that embraces the spectrum of sicca syndromes, incorporates advanced immunophenotyping, and allows inclusion of seronegative patients in research and care algorithms.

## 1. Introduction

Primary Sjögren’s syndrome (pSS), a chronic systemic autoimmune disease characterized by lymphocytic infiltration of the exocrine glands and frequently accompanied by systemic features and autoantibody positivity, is most frequently linked to Sicca syndrome, which is characterized by xerostomia (dry mouth) and keratoconjunctivitis sicca (dry eyes) [1]. However, a growing number of patients exhibit sicca symptoms without meeting the 2016 ACR/EULAR classification criteria for pSS and without the presence of the two main serological markers, anti-SSA (Ro) and anti-SSB (La) antibodies [2,3]. This patient population falls into a gray area in clinical immunology and rheumatology, which presents difficulties for researchers and clinicians in terms of diagnosis, prognosis, and treatment. This article presents a narrative review, synthesizing current knowledge on the clinical spectrum, immunologic findings, and therapeutic challenges of seronegative sicca syndrome. Given the heterogeneity of this underrecognized population and the limited controlled studies, a narrative format was selected in this paper to allow integration of emerging evidence and expert perspectives.

Although the criteria for classifying pSS have changed over the years, the importance of serological markers in these criteria frequently results in the exclusion of patients who lack detectable autoantibodies, despite manifesting classic symptoms and objective glandular dysfunction [2,4]. Such seronegative patients may experience a diagnostic journey in clinical practice, often being classified as having “undifferentiated sicca syndrome” or “incomplete Sjögren’s”, with little agreement on terminology, monitoring, or treatment approaches [5]. These patients are frequently understudied, underserved, and unclassified.

Patients with seronegative sicca are not all the same. While some may present with histology that is entirely nonspecific or even normal, others may exhibit histological findings suggestive of Sjögren’s disease, such as a positive minor salivary gland biopsy with focal lymphocytic sialadenitis and a focus score ≥ 1 [6,7]. While some patients may have systemic symptoms like fatigue, arthralgia, or neuropathy, the severity of glandular hypofunction varies, and these symptoms are not always enough to meet classification thresholds or to justify aggressive immunosuppressive therapy [8]. This clinical variation makes it even more difficult to comprehend the natural history and pathophysiology of seronegative sicca presentations.

Important concerns regarding the present shortcomings of our diagnostic instruments are brought up by the serological gap in these patients. Although anti-SSA and anti-SSB antibodies are a major component of conventional serologies, there is evidence that other immune dysregulations, both humoral and cellular, may also be at play [9,10]. It should not be assumed that autoimmune disease does not exist just because these particular autoantibodies are absent. Finding new immunological and genetic biomarkers that could aid in the stratification of seronegative patients and offer insight into possible disease mechanisms is gaining attention [11,12].

The long-term course of these patients is another issue. Some of them may develop full-blown Sjögren’s syndrome or another systemic autoimmune disease, while others may continue to have isolated sicca symptoms [13]. It is constantly difficult to predict which patients will take which course, and the absence of clear prognostic indicators contributes to uncertainty in clinical decision-making.

The purpose of this review is to summarize the information that is currently available regarding seronegative patients who exhibit symptoms of sicca but do not meet the requirements for Sjögren’s syndrome. We will look at the population’s epidemiology and demographics, clinical characteristics, immunological and histopathological study results, and potential pathophysiological mechanisms underlying their symptoms. The diagnostic challenges and possible overlaps with other rheumatic and non-rheumatic diseases will also be covered, along with treatment strategies and future research efforts targeted at enhancing outcomes for this underappreciated population.

This review promotes a more comprehensive understanding of dry syndrome that goes beyond conventional serological boundaries by pointing out the classification gaps and the new information about seronegative, non-Sjögren sicca presentations [10,14]. By doing this, we hope to provide insight into a subset of patients who might benefit from more specialized care and individualized treatment but are currently outside the spectrum of conventional diagnostic frameworks. 

**Figure 1:**
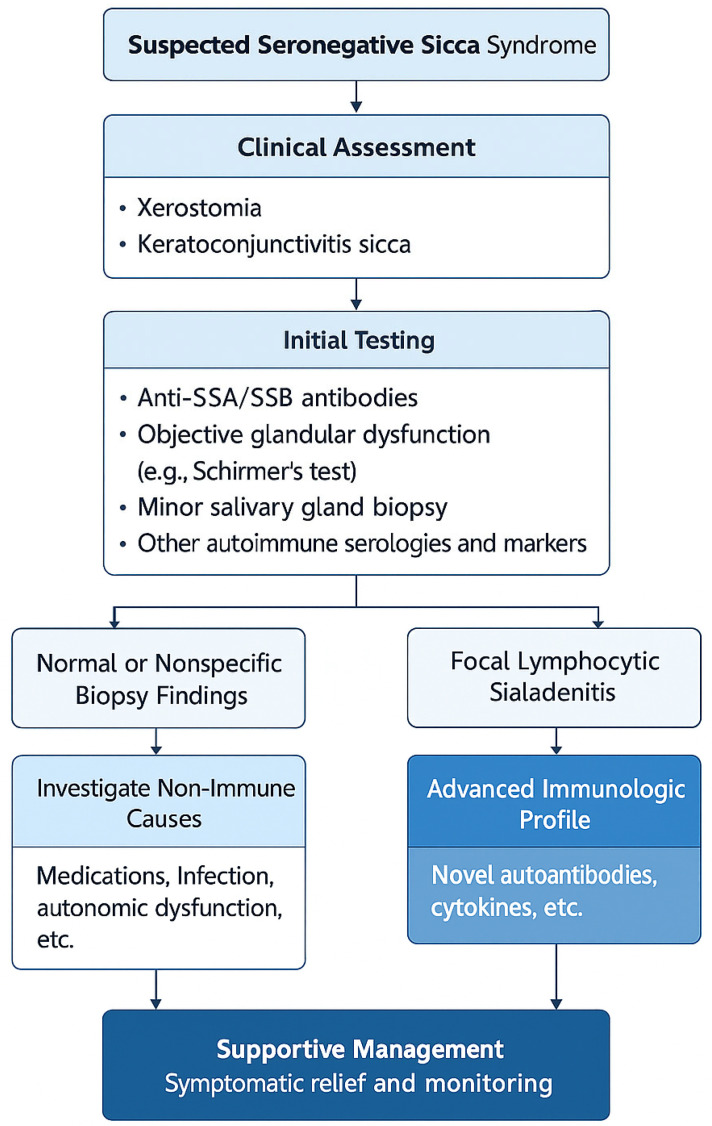
Proposed diagnostic framework for evaluating seronegative sicca syndrome. The chart guides clinicians from initial symptoms to biopsy findings and immunologic profiling.

Given diagnostic uncertainty regarding seronegative sicca syndrome, a working clinical definition is recommended encompassing sicca symptoms, negativity for anti-SSA/SSB, and not fulfilling ACR/EULAR classification criteria for pSS. They require separate consideration and follow-up. A new model for classification—perhaps “probable autoimmune exocrinopathy” or “non-criteria sicca syndrome”—is increasingly becoming a necessity. A systematic diagnostic approach is presented in Figure 1.

## 2. Epidemiology and Patient Demographics

The true epidemiological footprint of seronegative sicca syndrome remains elusive, largely due to the lack of standardized diagnostic criteria and the inherent bias in many cohort studies that require serological positivity as a condition for inclusion. As a result, patients with sicca symptoms who are seronegative for anti-SSA/Ro and anti-SSB/La antibodies—and do not fulfill the ACR/EULAR classification criteria for primary Sjögren’s syndrome—often go unreported in formal registries. Nonetheless, emerging studies suggest that this group represents a significant proportion of the broader sicca population [15].

Population-based estimates indicate that between 5% and 25% of patients presenting with dryness symptoms may fall into the seronegative category, depending on the criteria used and the population studied [16]. These patients may exhibit similar demographic trends as seropositive individuals, such as a marked female predominance and middle-age onset, but typically present with milder systemic involvement and lower autoimmune burden [17]. Reported prevalence varies by geographic region, methodology, and inclusion criteria (Table 1). For instance, European cohorts from Italy and Spain report that seronegative patients constitute up to one-third of referrals for sicca, whereas Asian studies (e.g., China) report lower rates, likely reflecting differences in healthcare access, genetics, and diagnostic practices. Age-stratified data indicate peak onset in the fifth and sixth decades, with strong female predominance. Importantly, many epidemiologic studies introduce selection bias by requiring autoantibody positivity for entry, underestimating the true burden of seronegative cases.

In various European cohorts, particularly from Italy and Spain, seronegative patients represented up to one-third of the total patients referred for suspected Sjögren’s, although their inclusion in formal registries was often inconsistent. Some studies, such as the one by Quartuccio et al., have shown that seronegative patients have a significantly lower prevalence of extraglandular manifestations and almost negligible lymphoma risk compared to their seropositive counterparts [5].

## 3. Clinical Characteristics and Symptom Spectrum

Seronegative sicca syndrome exhibits a wide range of clinical presentations, from isolated glandular dryness to mild systemic involvement that mimics early Sjögren’s syndrome. The cardinal symptoms include xerostomia and keratoconjunctivitis sicca, often associated with parotid enlargement and dental complications [18]. However, fatigue, arthralgia, and myalgia are also common, and may lead to diagnostic confusion with conditions such as fibromyalgia or undifferentiated connective tissue disease [19].

Despite the absence of anti-SSA/SSB antibodies, a subset of seronegative patients show evidence of immune dysregulation in other forms, such as polyclonal hypergammaglobulinemia, rheumatoid factor positivity, or mild cytopenias [20]. These laboratory features, although not specific, can serve as indirect markers of immune activity and support the autoimmune nature of the disease in the absence of classic serologies.

Studies have also reported that systemic manifestations in seronegative patients are usually less frequent and less severe compared to seropositive patients [5,17]. For example, interstitial lung disease, renal tubular acidosis, and peripheral neuropathy are relatively rare but can still occur, highlighting the need for careful clinical monitoring even in the seronegative population.

Interestingly, some seronegative patients fulfill all clinical and histologic features of Sjögren’s syndrome except for the serology component, suggesting that they may represent an earlier or milder phenotype of the disease rather than a separate entity [21]. These patients may progress slowly, remain stable, or convert to seropositive status over time, further complicating their classification and management.

A growing interest in objective measurement of symptom burden, particularly fatigue, has led to tools like the EULAR Sjögren’s Syndrome Patient Reported Index (ESSPRI), which may help stratify seronegative patients based on symptom severity even in the absence of systemic involvement [22].

## 4. Histopathology and Imaging

Minor salivary gland biopsy remains one of the most critical diagnostic tools in the evaluation of patients with suspected Sjögren’s syndrome, particularly in seronegative individuals. Focal lymphocytic sialadenitis (FLS) with a focus score ≥ 1 (defined as ≥1 lymphocytic aggregate per 4 mm^2^) is a key histological hallmark and carries significant diagnostic weight in the 2016 ACR/EULAR classification criteria [4].

In seronegative patients, biopsy findings are heterogeneous. Some show clear FLS with or without germinal center formation, while others demonstrate nonspecific chronic inflammation, fibrosis, or even normal histology [6,7]. This variability complicates the interpretation of biopsy results, particularly in patients without corroborative serology.

Immunohistochemical studies suggest that even in the absence of anti-SSA/SSB antibodies, there may be local B-cell hyperactivity, presence of follicular dendritic cells, and ectopic lymphoid neogenesis, supporting the autoimmune nature of disease in some seronegative cases [23].

Imaging modalities such as salivary gland ultrasonography (SGUS) and sialography can offer non-invasive assessment of glandular structure and function. SGUS, in particular, has shown promise in identifying hypoechoic areas and parenchymal inhomogeneity that correlate with histologic inflammation. However, the sensitivity and specificity of these tools in seronegative populations remain under evaluation (Table 2) [24].

## 5. Immunological Profile and Biomarkers

The seronegative status of many patients with sicca symptoms has historically led to the presumption that they are “non-autoimmune.” However, recent studies have challenged this binary categorization by uncovering a wide spectrum of immunologic alterations in seronegative individuals, including emerging biomarkers that fall outside the traditional anti-SSA/SSB panel [10,25].

Several promising autoantibodies have been identified in this population, such as anti-SP1 (salivary gland protein 1), anti-PSP (parotid secretory protein), and anti-carbonic anhydrase VI. These have shown potential utility in early disease detection, especially in patients who do not yet meet ACR/EULAR criteria [26]. However, their clinical use remains restricted to research settings due to limited commercial availability and lack of large-scale validation.

Other non-traditional immune markers such as anti-muscarinic type 3 receptor (M3R) antibodies have been linked to functional glandular impairment and autonomic dysfunction in both seronegative and seropositive patients. These antibodies are hypothesized to interfere directly with cholinergic signaling in salivary glands, contributing to xerostomia in the absence of structural damage [27].

Additionally, elevated levels of B-cell activating factor (BAFF), β2-microglobulin, and Fms-like tyrosine kinase 3 ligand (Flt3L) have been found in some seronegative patients, suggesting active immune stimulation even without classical autoantibodies [11,28,29].

Genomic studies using Mendelian randomization and plasma proteomics are also revealing candidate pathways and potential therapeutic targets, hinting that seronegative and seropositive disease may share underlying pathogenic architecture but diverge in surface expression [30,31]. 

The pathophysiology of seronegative sicca remains incompletely understood but appears to overlap with that of seropositive primary Sjögren’s syndrome in some cases. While classic Sjögren’s involves B-cell hyperactivity and autoantibody production, seronegative patients may exhibit more subtle T-cell-driven inflammation, local cytokine activation (e.g., BAFF, IFN-α), and neuroimmune dysregulation [32,33]. Histologic evidence of focal lymphocytic sialadenitis and ectopic lymphoid neogenesis supports an immune-mediated process, even in the absence of detectable autoantibodies [32]. In other patients, non-immune mechanisms such as autonomic dysfunction or acinar cell apoptosis may predominate. The heterogeneity suggests the existence of multiple endotypes under the umbrella of seronegative sicca.

## 6. Differential Diagnosis

The evaluation of patients with sicca symptoms and negative serology necessitates a broad and systematic differential diagnosis (Table 3). Not all patients with dry eyes and dry mouth have an underlying autoimmune disorder, and distinguishing between true immunologic sicca and other mimics is essential for accurate classification and appropriate treatment [34].

Age-related glandular atrophy is one of the most common non-pathological causes of dryness, particularly in elderly patients. It is characterized by reduced acinar cell density and diminished salivary flow without evidence of inflammation or autoimmunity.

Medication-induced sicca is another frequent cause, especially in polypharmacy settings. Common culprits include anticholinergics, antihistamines, antidepressants, and diuretics. A careful medication history is often sufficient to identify drug-induced symptoms, which may reverse with withdrawal of the offending agent [35].

Fibromyalgia and chronic fatigue syndrome may present with prominent dryness and fatigue, leading to diagnostic confusion. However, these syndromes typically lack objective evidence of glandular dysfunction or immunologic activity.

Viral infections, particularly hepatitis C virus (HCV), HIV, and HTLV-1, can cause sicca-like symptoms and may even be accompanied by positive autoantibodies. Hepatitis C-related sicca may mimic pSS, and distinction requires viral serology and assessment of liver involvement [36].

IgG4-related disease can affect the salivary and lacrimal glands and present with painless swelling and xerostomia. Histopathology in these cases reveals dense lymphoplasmacytic infiltrates with IgG4-positive plasma cells and storiform fibrosis.

Other conditions to consider include sarcoidosis, amyloidosis, diabetes mellitus, and endocrinopathies such as hypothyroidism. In some cases, overlapping features may exist, requiring multidisciplinary evaluation.

## 7. Prognosis and Disease Evolution

The long-term outcomes of patients with seronegative sicca syndrome are variable and remain poorly defined. Unlike their seropositive counterparts, who have been extensively studied through large registries and clinical trials, seronegative patients are often excluded from long-term follow-up cohorts, leaving major gaps in our understanding [37].

Some individuals with isolated glandular symptoms may remain stable for years without developing systemic features or seroconversion. In such cases, the prognosis is generally favorable, and symptomatic treatment focused on dryness management may suffice [38]. However, a subset of patients may eventually fulfill the classification criteria for pSS through either the emergence of systemic manifestations or seroconversion—particularly for anti-SSA antibodies—after several years of disease [39].

Factors associated with disease progression are not well established. Preliminary data suggest that the presence of certain histopathologic features (e.g., high focus score or germinal centers), elevated BAFF or β2-microglobulin levels, or subtle systemic complaints such as neuropathy or arthritis may herald a more active or evolving disease phenotype [11,28].

Importantly, the risk of lymphoma, a major concern in seropositive pSS, appears to be significantly lower in seronegative patients. Quartuccio et al. and others have reported a nearly negligible lymphoma rate in patients lacking SSA/SSB antibodies and systemic activity [5]. This observation may reflect lower B-cell clonal expansion or simply less immune dysregulation overall.

Nonetheless, the unpredictability of disease course—ranging from complete stability to insidious progression—highlights the need for regular follow-up. Even in apparently stable patients, evolving features such as leukopenia, glandular swelling, or unexplained fatigue should prompt a reevaluation of the diagnosis and closer surveillance [40].

To date, no validated prognostic score exists for seronegative sicca patients, and efforts are needed to stratify risk based on clinical, immunologic, and histologic profiles—perhaps modeled after existing tools like the ESSDAI (EULAR Sjögren’s Syndrome Disease Activity Index), which remains underused in this population. 

A key consideration in the follow-up of seronegative sicca patients is the potential for seroconversion, particularly the later emergence of anti-SSA (Ro) antibodies. Although the majority of patients remain seronegative and clinically stable over time, longitudinal studies suggest that approximately 5–10% may seroconvert after several years, often alongside the development of systemic manifestations. This highlights the importance of ongoing surveillance even in initially mild cases [5,13].

Certain features may help identify individuals at higher risk of seroconversion or disease evolution. These include minor salivary gland biopsy showing focal lymphocytic sialadenitis with a focus score ≥ 1, early systemic symptoms (e.g., fatigue, arthralgia, neuropathy), and laboratory indicators such as polyclonal hypergammaglobulinemia, cytopenias, or elevated inflammatory markers [5,13]. Additionally, elevated levels of BAFF or β2-microglobulin, and the presence of non-classical autoantibodies (e.g., anti-SP1, CA-VI) may indicate latent immune dysregulation in seronegative individuals. Patients with such findings should undergo regular clinical and immunologic reassessment, as they may eventually meet classification criteria for primary Sjögren’s syndrome or another systemic autoimmune disease.

A conceptual continuum exists between non-immune and immune-mediated forms of sicca. At one end are functional or age-related dryness, and at the other, fully classified primary Sjögren’s syndrome. In between lie seronegative sicca, incomplete Sjögren’s, and syndromes with overlapping features such as fibromyalgia or chronic fatigue syndrome, which may present with similar symptom burdens but lack objective glandular dysfunction or histopathologic evidence of autoimmunity. Differentiating these entities requires a combination of symptom scoring, imaging, biopsy, and emerging biomarkers.

## 8. Therapeutic Strategies and Unmet Needs

Treatment of seronegative sicca patients remains largely empirical due to their exclusion from most therapeutic trials and the lack of validated treatment algorithms specific to this group (Table 4). As a result, management often borrows from the principles used in seropositive primary Sjögren’s syndrome, despite clear differences in pathophysiology and prognosis [38].

For patients with isolated glandular symptoms, first-line therapy includes symptomatic management such as artificial tears, saliva substitutes, and meticulous dental care. Pilocarpine or cevimeline—cholinergic agonists—can be used to stimulate residual glandular function, although these may be less effective in advanced glandular atrophy [39].

In patients with fatigue, arthralgia, or other mild systemic symptoms, hydroxychloroquine is commonly used, despite limited evidence of efficacy in seronegative individuals. Fatigue in particular is often multifactorial, and non-pharmacologic approaches such as cognitive behavioral therapy, graded exercise, and sleep hygiene should be emphasized [22].

Immunosuppressive therapies such as methotrexate, azathioprine, or mycophenolate mofetil are rarely indicated and typically reserved for patients with biopsy-proven inflammation and significant extraglandular involvement. Given the overall lower risk of systemic complications and lymphoma in seronegative patients, aggressive immunosuppression is generally not warranted [5].

There is also growing interest in B-cell–targeted therapies like rituximab, which have shown some efficacy in pSS but remain unproven in seronegative disease. In the absence of classic B-cell hyperactivity markers (e.g., anti-SSA, hypergammaglobulinemia), their benefit is questionable and should be considered on a case-by-case basis [40].

Emerging biologics targeting BAFF, IFN pathways, and TLR signaling are being evaluated in clinical trials for pSS, but none have been tested in a seronegative cohort. Personalized immunologic profiling could help identify subgroups of seronegative patients who might benefit from such approaches, representing a major unmet need in this population [30].

New composite measures such as STAR (Sjögren’s Tool for Assessing Response) and CRISS (Composite of Relevant Immune Sjögren’s Symptoms) are under development to better quantify both patient-reported outcomes and objective inflammation in trials [41,42]. While these tools are still being validated, they may be particularly useful in characterizing response in heterogeneous or seronegative populations.

In terms of emerging therapies, biologics beyond rituximab have gained attention. Iscalimab, a monoclonal antibody targeting CD40, has shown encouraging results in early-phase trials by modulating T-cell–B-cell interaction [43]. Although data are still limited, such agents may offer future options for patients with seronegative disease who demonstrate immunologic activity.

## 9. Discussion

The exploration of seronegative sicca syndrome—suffered by patients who present with xerostomia and/or keratoconjunctivitis sicca but lack anti-SSA/SSB antibodies and do not fulfill current classification criteria for primary Sjogren’s syndrome [2] (pSS)—reveals a clinical domain that is at once familiar and enigmatic. While such patients often resemble those with pSS in their symptomatology, they inhabit a diagnostic gray zone that challenges the current paradigms of autoimmunity, classification, and care delivery.

## 10. Redefining Disease Boundaries

One of the central questions raised by this review is whether seronegative sicca syndrome represents a milder, early, or incomplete form of pSS, or whether it constitutes an entirely separate clinical entity. The answer is unlikely to be uniform across all cases. Evidence suggests that a spectrum exists—from patients with subclinical or evolving autoimmune disease, to those with idiopathic, non-immune exocrine gland dysfunction.

Histologic studies have shown that a proportion of seronegative patients have focal lymphocytic sialadenitis with elevated focus scores, similar to those with classified pSS [6], indicating that immune-mediated glandular damage can occur even in the absence of serologic markers. Others show normal or nonspecific histology, pointing instead to functional, age-related, or environmental causes of dryness. Imaging via salivary gland ultrasonography adds another layer [24] of complexity, sometimes revealing abnormalities that parallel those seen in seropositive patients.

The clinical heterogeneity of this population makes the binary classification of “Sjogren’s” vs. “non-Sjogren’s” inadequate for capturing disease reality. Recent efforts using high-throughput and single-cell technologies have proposed the existence of distinct phenotypic clusters within primary Sjögren’s syndrome. These include glandular-dominant, systemic, and neuropathic subtypes, each potentially driven by different immunopathologic mechanisms [44]. Such stratification may offer a more individualized approach to both classification and therapy, particularly relevant in seronegative patients who do not fit neatly into current diagnostic frameworks. The current criteria are primarily designed for clinical trials and epidemiological uniformity, not for nuanced diagnosis or therapeutic guidance. As such, many patients with genuine disease burden remain “unclassified”, lacking both a diagnosis and a path forward.

## 11. Immune Activity Beyond Serology

Traditional autoantibodies—anti-SSA, anti-SSB, ANA, and RF—are heavily weighted [1,2] in classification, but emerging evidence suggests that seronegative patients may still exhibit immune dysregulation. Elevated cytokines (e.g., BAFF, IFN-alpha), abnormal T-cell subsets, and alternative autoantibodies (e.g., SP-1, CA-VI [10,11,26]) support the notion that the immune system is active in at least a subset of these patients. The absence of conventional serologic markers may reflect limitations in our current tools, rather than true immunological silence.

The discovery of novel biomarkers and immune signatures is therefore essential. These could not only refine diagnosis but also predict disease evolution and treatment response. This is especially pertinent given that some seronegative patients eventually seroconvert [37] or develop systemic features over time. Identifying these individuals early could allow for targeted monitoring and preemptive intervention.

## 12. Clinical Implications and Patient Burden

From a clinical standpoint, one of the most pressing challenges is the disconnect between classification and care. Patients with seronegative sicca syndrome often experience substantial symptom burden—dryness, fatigue, pain, and functional limitations—yet face barriers to diagnosis, therapy, and support. They may be told that “nothing is wrong” due to negative tests, leading to frustration, psychological distress, and reduced engagement with the healthcare system.

Moreover, these patients are largely excluded from clinical trials, registries, and therapeutic innovation [16,19]. They fall outside the scope of most treatment algorithms, and decisions are often made on a case-by-case basis, without guidance from evidence-based protocols. This therapeutic problem disproportionately affects women, older adults, and those with comorbid conditions such as fibromyalgia [22], further widening gaps in care.

The need for individualized, symptom-based management [36] is clear. While immunosuppressive therapies may not be appropriate for all, the use of local treatments, lifestyle strategies, and multidisciplinary support [27] can offer meaningful relief. Additionally, trials that include or specifically target seronegative populations are urgently needed to generate applicable data and reduce therapeutic inequities.

## 13. Bridging Gaps in Research and Practice

In light of these considerations, a paradigm shift is needed-away from a rigid, antibody-centric view of disease, and toward a more holistic and inclusive approach. This includes the following:

- Embracing the spectrum model of sicca syndromes, with varying degrees of immune involvement;
- Developing new diagnostic categories such as “probable autoimmune exocrinopathy” or “non-criteria sicca syndrome”;
- Incorporating biomarker discovery and advanced immunophenotyping into routine research;
- Ensuring clinical trial access for seronegative patients;
- Validating patient-reported outcomes as core endpoints in future studies.

Historically, efforts to classify patients with sicca symptoms have not always relied on the presence of autoantibodies. Pioneering work by Moutsopoulos and colleagues emphasized the significance of clinical presentation and long-term disease trajectory in defining Sjögren’s syndrome, particularly in patients lacking conventional serologic markers [45]. In a landmark study, they described a substantial subset of patients with sicca features, objective glandular dysfunction, and lymphocytic infiltration on biopsy, yet without anti-SSA/SSB antibodies, who nonetheless shared disease behavior with seropositive individuals. Their approach—grounded in the evaluation of symptom burden, biopsy findings, and systemic involvement—highlights the necessity of a broader clinical lens when evaluating seronegative cases.

This clinical model aligns with the perspective advanced in the current review: that rigid serology-based classification risks overlooking genuine autoimmune pathology. Incorporating both clinical and immunologic parameters—such as fatigue, biopsy-confirmed sialadenitis, and emerging biomarkers—into a more inclusive framework could bridge the divide between empirical rheumatology practice and narrowly defined research criteria.

## 14. Conclusions

Seronegative sicca syndrome—defined by the presence of dry mouth and/or dry eyes in the absence of anti-SSA/SSB antibodies and without fulfillment of current classification criteria for primary Sjögren’s syndrome—represents a clinically significant but poorly characterized domain within rheumatology and immunology. These patients challenge the traditional boundaries of autoimmune disease and highlight the limitations of serology-driven diagnosis.

This review underscores the heterogeneity of the seronegative sicca population. While some individuals exhibit subtle immune dysregulation, focal lymphocytic sialadenitis, or systemic features that suggest early or incomplete autoimmunity, others appear to have functional, age-related, or iatrogenic glandular dysfunction. Importantly, seronegativity does not equate to the absence of disease, nor does it negate the real burden experienced by patients in terms of dryness, fatigue, and diminished quality of life.

Current classification systems, heavily reliant on autoantibodies and invasive diagnostics, often fail to accommodate these individuals, leaving them at a diagnostic and therapeutic crossroad. This has profound consequences for access to care, research participation, and long-term monitoring. The lack of inclusion in clinical trials further widens the gap between patient needs and available evidence.

There is an urgent need for more inclusive and nuanced frameworks that recognize the spectrum of sicca presentations. Expanded biomarker panels, imaging modalities, and immunologic profiling could help stratify patients more accurately, allowing for tailored management and meaningful prognostication. Longitudinal cohort studies will be essential to understanding the natural history of this group and identifying predictors of progression or stability.

From a therapeutic standpoint, individualized care remains paramount. While immunosuppressive therapy is not universally indicated, symptomatic treatment, lifestyle modifications, and multidisciplinary support can greatly enhance function and well-being. As our understanding deepens, future therapeutic strategies should be built not only on immunologic stratification but also on patient-reported outcomes that reflect real-life burdens.

Ultimately, recognizing and validating the experiences of seronegative sicca patients is both a clinical necessity and an ethical imperative. These patients remind us that not all disease is captured by laboratory tests—and that attentive listening, thorough evaluation, and inclusive science must remain central to our approach to care.

## 15. Clinical Take-Home Points

- Not all patients with sicca symptoms and immune activation meet criteria for primary Sjögren’s syndrome—seronegative sicca represents a distinct and underdiagnosed entity.
- A subset of seronegative patients may eventually seroconvert or develop systemic features; longitudinal follow-up is essential.
- Histopathologic findings and emerging biomarkers (e.g., BAFF, SP1) can provide critical clues to immune involvement in the absence of anti-SSA/SSB.
- Management should be individualized, balancing symptom relief with careful immune profiling before considering immunosuppression.
- Inclusion of seronegative patients in trials and classification updates is essential to close care gaps and reflect real-world diversity.

## Figures and Tables

**Table 1:** Demographic features of seronegative sicca patients across studies.

Study (Author, Year)	Country	Sample Size	% Female	Mean Age (years)	Anti-SSA/SSB Negative (%)	Systemic Symptoms (%)	Key Notes
Quartuccio et al. [5], 2015	Italy	133	91%	56.4	100%	24%	Lower lymphoma risk
Ramos-Casals et al. [13], 2008	Spain	1010	94%	54.2	~25%	32%	Registry data
Tu et al. [17], 2022	China	1 (case)	Female	42	100%	Hypokalemia	Case report
Acharya et al. [18], 2023	Nepal	1 (case)	Female	38	100%	Biliary overlap	Rare combo
Maripuri et al. [6], 2009	USA	25	88%	55.7	100%	Renal involvement	Biopsy cohort

**Table 2:** Histopathological and imaging findings in seronegative vs. seropositive patients.

Feature	Seronegative Patients	Seropositive Patients
Focus Score ≥ 1	~40–60% (variable)	~80–90%
Germinal Centers	Rare (<10%)	Common (~30–40%)
Fibrosis	More prevalent	Less common
SGUS (hypoechoic areas)	Moderate sensitivity	High sensitivity
Biopsy-negative with symptoms	Frequent (~30–40%)	Rare (<10%)
Use in classification criteria	Often decisive	Often corroborative

**Table 3:** Differential diagnosis of seronegative sicca syndrome.

Condition	Key Features	Suggested Approach
Age-related dryness	Older adults, slow onset, no inflammation	Unremarkable labs, normal biopsy
Medication-induced sicca	Linked to drug use	Discontinue meds, reassess symptoms
Fibromyalgia/CFS	Fatigue, pain, normal labs	Clinical diagnosis, no glandular damage
Hepatitis C–related sicca	Positive RF, arthralgia, liver enzymes	HCV serology, liver ultrasound
IgG4-related disease	Painless gland swelling	IgG4 levels, biopsy with IgG4 stain
Sarcoidosis	Parotid swelling, hilar lymphadenopathy	Chest X-ray, CT, MRI, biopsy
Diabetes, hypothyroidism	Metabolic symptoms	Glucose, TSH, antithyroid antibodies

**Table 4:** Therapeutic approaches in seronegative sicca patients by clinical profile.

Clinical Profile	First-Line Treatment	Optional/Second-Line	Notes
Isolated sicca (glandular only)	Saliva/tear substitutes, pilocarpine	Cevimeline	Focus on symptomatic relief
Fatigue, arthralgia (no systemic damage)	Hydroxychloroquine, lifestyle	Cognitive Behavioral Therapy, low-dose antidepressants	Screen for fibromyalgia
Biopsy-proven FLS, mild extraglandular	Hydroxychloroquine	Methotrexate or low-dose steroids	Confirm diagnosis before escalation
Significant systemic involvement	Not typical	Case-by-case basis, consider MTX/MMF	Rare in true seronegative patients
Refractory symptoms, autoantibody-negative	Limited evidence	Experimental biologics (rituximab, BAFF-i)	Consider trial enrollment
